# The Largest Number Representable in 64 Bits

**DOI:** 10.3390/e28050494

**Published:** 2026-04-26

**Authors:** John Tromp

**Affiliations:** Independent Researcher, 1111CP Diemen, The Netherlands; john.tromp@gmail.com

**Keywords:** busy beaver, Turing machine, lambda calculus, Kolmogorov complexity, Algorithmic Information Theory

## Abstract

We investigate how large an output can be computed by programs fitting inside a single register, using languages not designed for generating large outputs. We propose lambda calculus-based Busy Beaver functions that offer various advantages over the existing Turing machine-based ones, including a direct relation to Kolmogorov complexity.

## 1. Introduction

Most people believe $2^{64}-1=18,446,744,073,709,551,615$, or FFFFFFFFFFFFFFFF in hexadecimal, to be the largest number representable in 64 bits. In English, it is quite the mouthful: eighteen quintillion four hundred and forty-six quadrillion seven hundred and forty-four trillion seventy-three billion seven hundred and nine million five hundred and fifty-one thousand six hundred and fifteen.

That is indeed the maximum possible value of 64 bit-unsigned integers, available as data type uint64_t in C or u64 in Rust. Floating point data types can represent much larger values, courtesy of their base 2 exponent. The 64-bit floating point standard known as IEEE-754, available as data type double in C or f64 in Rust, has a largest (finite) representable value of $2^{1024}\left(1-2^{-53}\right)\approx 1.8\times 10^{308}$.

But we need not limit ourselves to plain data types. What if we allow richer types of representations? Since we want representations to remain computable, the most general kind of representation would be (the output of) a program in some programming language. But the program must be small enough to fit in 64 bits. Our quest then will be for the largest number programmable in 64 bits.

## 2. Small Programs

Some programming languages are not suitable for this task, due to required scaffolding. A popular language like C, for instance, while sporting the famous IOCCC (International Obfuscated C Code Contest) featuring small programs doing unbelievable things, requires every valid program to declare the main function such as main(){}, consisting of eight ASCII characters. While technically, ASCII is a 7-bit character encoding standard representing 128 unique characters, all modern computers use 8-bit bytes to store either plain ASCII or UTF-8, a Unicode character encoding that is backward compatible with ASCII. So main(){} is essentially the only valid 64-bit C program.

Plenty other languages require no such scaffolding. For instance, Linux features the arbitrary precision calculator bc, which happily evaluates programs like 9^999999 to the 954,242 digit number 35,908,462…48,888,889, making it programmable in 8 bytes. So is the much larger 9^9^9^99 $=9^{9^{9^{99}}}$ with over $10^{10^{94}}$ digits, which bc is less happy to compute. If bc supported the symbol ! for computing factorials, then 9!!!!!!! would represent a much larger number still.

## 3. To Cheat or Not to Cheat

Allowing such primitives feels a bit like cheating though. Would we allow a language that has the Ackermann function predefined, letting the 8 byte expression ack(9,9) represent a truly huge number?

Many people will be quick to dismiss our quest entirely, arguing that whatever number N may be the largest representable in 64 bits, they can propose a new language in which the 1-bit program 0 is defined to “evaluate” to N+1. That is a clear example of cheating, i.e., designing a language to get the results you want. Those people may then also ask exactly what qualifies as non-cheating, but this is something that cannot be formalized, only recognized after the fact.

Our quest is for the largest number programmable in 64 bits without cheating. It is precisely our self-imposed program size limit that necessitates considerations of cheating. If we are only concerned with asymptotic behavior, then cheating ceases to be a concern, as we will see in Section 12.

## 4. Ackermann Considered Unhelpful

As it turns out, the question of whether Ackermann is allowed, is moot. One can blow way past ack(9,9) in under 64 bits in a language with no built-in primitive whatsoever. A language with no basic arithmetic; not even numbers themselves. A language in which all those must be defined from scratch.

But let us first look at another language lacking the usual programming primitives, one that has been particularly well studied for producing largest possible outputs. That is the language of Turing machines.

## 5. The Busy Beaver

The famous Busy Beaver function, introduced by Tibor Radó in 1962 [1], which we will denote BB(*n*), is defined as the maximal number of steps taken by an *n*-state Turing Machine (TM) with a binary tape alphabet, starting from an all 0 tape, before halting.

Here we have a discrepancy between how the size of a TM is measured, in states, versus how program size is measured, in bits. Fortunately there is a straightforward binary encoding of *n*-state TMs, which is entirely determined by its transition function. For each of the *n* states that the machine’s finite control can be in, and each of its two tape symbols that could be scanned by its tape head, the transition function specifies what new symbol to write in the scanned tape cell (1 bit), whether to move the tape head left or right (1 bit), and what new state (or special halt state) to transition to ($\left\lceil \log_{2}\left(n+1\right)\right\rceil$ bits). This encoding takes 6×2×(2+3)=60 bits for a 6-state TM, and 7×2×(2+3)=70 bits for a 7-state TM.

We are also stretching the meaning of “representable” a bit since BB considers the runtime of the machine instead of its output. Besides the above BB (that Radó called S), Radó also defined another function called Σ that considers the output of the machine as a number in unary, i.e., the number of 1s in the final tape contents. But BB has received more attention, as it allows one to determine from BB(*n*) all halting *n*-state machines. For 6-states and up though, there is no discernible difference in magnitude between the two functions (even an exponential difference fades between tetrational values) so we could have just as easily used Σ.

So the largest number TM programmable in 64 bits is BB(6).

## 6. How Large Is BB(6)?

Unfortunately, we may never know. While BB(*n*) has been determined (and even formally proven) for all n≤5 [2], there are some 6-state TMs whose halting behavior is closely related to very hard mathematical problems. Most of these so-called cryptids are likely not to halt, while some, like one called “Lucy’s Moonlight”, are likely to halt but unlikely to beat the current champion.

The current 6-state champion shows that BB(6)>2↑↑2↑↑2↑↑10. Here, m↑↑n is Knuth’s up-arrow notation for an exponential tower of *n m*’s, so that for example $2↑↑3=2^{2^{2}}$. As large as this number is, it is still very small compared to ${\mathrm{ack}\left(9,9\right)=2↑}^{7}12-3=2↑↑↑↑↑↑↑12-3$, where ${m↑}^{k+1}n$ denotes ${m↑}^{k}\left(m{↑}^{k}\ldots \left(m{↑}^{k}m\right)\ldots \right)$ with *n m*s.

It is known, however, that ${BB\left(7\right)>2↑}^{11}{2↑}^{11}3>\mathrm{ack}\left(9,9\right)$. Several leading BB researchers believe that BB(7) is even larger than the famous Graham’s Number, which iterates the function (${n\mapsto 3↑}^{n}3$) 64 times starting from n=4. This is a rather bold belief, considering that the smallest known Graham exceeding TM has twice as many states (14)—bold enough for me to offer a $1000 bet that a proof of BB(7) > Graham’s Number will not be found within 10 years, which leading BB researcher Shawn Ligocki was happy to accept.

Meanwhile, Graham’s Number is easily surpassed within 64 bits, by moving beyond Turing machines.

## 7. Lambda Calculus

Alonzo Church conceived the λ-calculus in 1932 [3] as a formal logic system for expressing computation based on function abstraction and application using variable binding and substitution.

The Graham beating lambda term originates in a Code Golf [4] challenge asking for the “Shortest terminating program whose output size exceeds Graham’s number”, answered by user Patcail and further optimized by user 2014MELO03. The following 49-bit program

0100011010000110011000000101101100000011100111010

is the Binary Lambda Calculus [5] encoding of the term

(λ11)(λ1(1(λλ12(λλ2(21)))))

where λ (lambda) denotes an anonymous function, and number *i* is the variable bound by the *i*-th nested λ. This is known as De Bruijn notation, a way to avoid naming variables. A more conventional notation using variable names would be

(λJ.JJ)(λy.y(y(λgλm.mg(λfλx.f(fx)))))

The last 16 bits of the program, making up almost a third of its size, encodes the term λfλx.f(fx), which takes arguments *f* and *x* in turn, and iterates *f* twice on *x*. In its generalized form, the function $\lambda f\lambda x.f^{n}x$, called Church numeral *n*, is the most common way of representing natural numbers in the λ-calculus. The encoding $\hat{n}$ of Church numeral *n* is $0000{\left(01110\right)}^{n}10$, of size 5n+6 bits. The program, which we will name after its discoverer, can be expressed more legibly as


Melo=let{2=λfλx.f(fx);H=λgλm.mg2;J=λy.y(yH)}inJJ

Melo evaluates to a Church numeral, “Melo’s Number”, that comfortably exceeds Graham’s Number, as we will prove after three supporting Lemmas.

**Lemma** **1.** *JJ=2↑↑6(HH)2*.

The following proof is formatted to show the redex on the left and unaffected arguments on the right.

**Proof.**  $\begin{array}{cccc} J\;J= & J\;(J\;H)=J\;(H\;(HH)) & & \\ = & H\;(HH)\;(H\;(HH)\;H) & & \\ = & H\;(HH)\;H\; & (HH)\;2 & \\ = & H\;(HH)\;2\; & (HH)\;2 & \\ = & 2\;(HH)\;2\; & (HH)\;2 & \\ = & HH\;(HH\;2) & (HH)\;2 & \\ = & HH\;2\; & H\;2\;(HH)\;2 & \\ = & 2\;H\;2\; & H\;2\;(HH)\;2 & \\ = & H\;(H\;2)\;H\; & 2\;(HH)\;2 & \\ = & H\;(H\;2)\;2\; & 2\;(HH)\;2 & \\ = & 2\;(H\;2)\;2\; & 2\;(HH)\;2 & \\ = & H\;2\;(H\;2\;2)\; & 2\;(HH)\;2 & \\ = & H\;2\;2\; & 2\;2\;2\;(HH)\;2 & \\ = & 2\;2\;2\; & 2\;2\;2\;(HH)\;2 & \\ = & 2↑↑6\;(HH)\;2 & & \end{array}$□

**Lemma** **2.** *For k,n≥2, ${k\;H\;2\;n>3↑}^{k}\left(1+n\right)$*.

**Proof.**  By induction on *k*.

Base: Since $H\;2\;\left(2↑↑i\right)=\left(2↑↑i\right)\;2\;2=2^{2^{2↑↑i}}=2↑↑\left(i+2\right)$,

$2\;H\;2\;n=H\;\left(H2\right)\;n=n\;\left(H2\right)\;2={2↑↑\left(1+2n\right)>3↑}^{2}\left(1+n\right)$ already at n=2, since $2↑↑5=2^{2^{16}}>3^{27}=3↑↑3$.

Step: $\left(k+1\right)\;H\;2\;n=H\;\left(k\;H\;2\right)\;n=n\;\left(k\;H\;2\right)\;2\overset{Lm\;2}{>}{3↑}^{k}\left(1+\;3{↑}^{k}\left(1+\ldots 3{↑}^{k}\left(1+2\right)\ldots \right)\right){>3↑}^{k}{3↑}^{k}\ldots 3{{↑}^{k}3=3↑}^{k+1}\left(1+n\right)$. □

**Lemma** **3.** *For n≥2, ${HH\left(HH\;n\right)>3↑}^{n}3$*.

**Proof.**  Lemma 1’s proof shows $HH\left(HH\;2\right)={2↑↑6>3↑}^{2}3$. Now suppose m=n−1≥2. Then $HH\;\left(HH\;n\right)=HH\;n\;H\;2=\left(1+m\right)\;H\;2\;H\;2=H\;\left(m\;H\;2\right)\;H\;2=H\;\left(m\;H\;2\right)\;2\;2=2\;\left(m\;H\;2\right)\;2\;2=m\;H\;2\;\left(m\;H\;2\;2\right)\;2\overset{Lm\;2}{>}{3↑}^{m}\left(1+3{↑}^{m}\left(1+2\right)\right){\;2>(3↑}^{m}{3↑}^{m}3{)\;2=2^{{3↑}^{n}3}>3↑}^{n}3$. □

**Theorem** **1.** *Melo $>Graham^{\prime }sNumberG\left(64\right)$, where $G\left(n\right)=n\left(n\mapsto 3{↑}^{n}3\right)4$*.

**Proof.**  Melo = $J\;J\overset{Lm\;1}{=}2↑↑6\;\left(HH\right)\;2\overset{Lm\;3}{>}\left(2↑↑6/2\right)\;\left(n\mapsto 3{↑}^{n}3\right)\;2=\left(2↑↑6/2-1\right)\;\left(n\mapsto 3{↑}^{n}3\right)\;\left(3{↑}^{2}3\right)>\left(2↑↑6/2-1\right)\;\left(n\mapsto 3{↑}^{n}3\right)\;4=G\left(2↑↑6/2-1\right)>G\left(64\right)$. □

## 8. Leaving Melo’s Number in the Dust

With 64−49=15 bits to spare, opportunities for vastly boosting Melo abound. Discord users 50_ft_lock and Sam found the following term that extends Melo’s H with an extra argument:

ω218=let {2=λfλx.f(fx);A=λaλbλc.cab2;T=λy.y(yA)} in TTT

which desugars to lambda term

(λT.TTT)(λy.y(y(λaλbλc.cab(λfλx.f(fx)))))

in conventional notation, or

(λ111)(λ1(1(λλλ132(λλ2(21)))))

in De Bruijn notation, with 61-bit encoding


0100010110101000011001100000000101011011101100000011100111010.

Illustrated below are Melo and ω218 as so-called lambda diagrams [6].

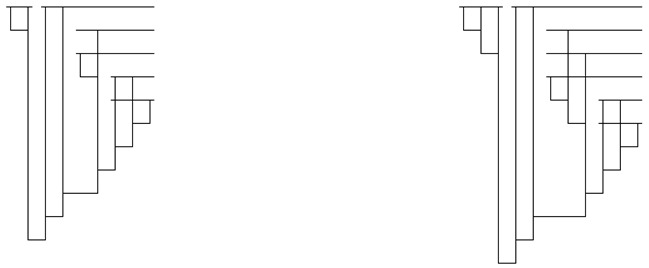



Let us first establish an analogue of Lemma 1:

**Lemma** **4.** *TTT=2↑↑18A2222222222*.

The following reduction was verified with the program symbolic.Blc featured in Section “Symbolic Lambda Calculus reduction” of my 2012 IOCCC entry [7].

**Proof.**  $\begin{array}{cccc} T\;T & T & & \\ = & T\;(T\;A) & T & \\ = & T\;(A\;(AA)) & T & \\ = & A\;(AA)\;(A\;(AA)\;A)\;T & & \\ = & T\;(AA) & (A(AA)A)2 & \\ = & AA\;(AA\;A)\;(A\;(AA)\;A) & 2 & \\ = & A\;(AA)\;A\;A & (AAA)22 & \\ = & A\;(AA)\;A\;2 & (AAA)22 & \\ = & 2\;(AA)\;A & 2(AAA)22 & \\ = & AA\;(AA\;A)\;2 & (AAA)22 & \\ = & 2A\;(AA\;A) & 2(AAA)22 & \\ = & A\;(A\;(AA\;A))\;2\;(AA\;A) & 22 & \\ = & AA\;A\;(A\;(AA\;A)) & 2222 & \\ = & A\;(AA\;A)\;A\;A & 22222 & \\ = & A\;(AA\;A)\;A\;2 & 22222 & \\ = & 2\;(AA\;A)\;A & 222222 & \\ = & AA\;A\;(AA\;A\;A) & 222222 & \\ = & AA\;A\;A & AA2222222 & \\ = & A\;A\;A\;2 & AA2222222 & \\ = & 2\;A\;A & 2AA2222222 & \\ = & A\;(AA)\;2\;A & A2222222 & \\ = & A\;(AA)\;2\;2 & A2222222 & \\ = & 2\;(AA)\;2 & 2A2222222 & \\ = & AA\;(AA\;2)\;2 & A2222222 & \\ = & 2\;A\;(AA\;2) & 2A2222222 & \\ = & A\;(A\;(AA\;2))\;2\;A & 2222222 & \\ = & A\;(A\;(AA\;2))\;2\;2 & 2222222 & \\ = & 2\;(A\;(AA\;2))\;2 & 22222222 & \\ = & A\;(AA\;2)\;(A\;(AA\;2)\;2)\;2 & 2222222 & \\ = & 2\;(AA\;2)\;(A\;(AA\;2)\;2) & 22222222 & \\ = & AA\;2\;(AA\;2\;(A\;(AA\;2)\;2)) & 22222222 & \\ = & AA\;2\;(A\;(AA\;2)\;2) & A2222222222 & \\ = & A\;(AA\;2)\;2\;A & 22A2222222222 & \\ = & A\;(AA\;2)\;2\;2 & 22A2222222222 & \\ = & 2\;(AA\;2)\;2 & 222A2222222222 & \\ = & AA\;2\;(AA\;2\;2) & 222A2222222222 & \\ = & AA\;2\;2 & A22222A2222222222 & \\ = & 2\;A\;2 & 2A22222A2222222222 & \\ = & A\;(A\;2)\;2\;A & 22222A2222222222 & \\ = & A\;(A\;2)\;2\;2 & 22222A2222222222 & \\ = & 2\;(A\;2)\;2 & 222222A2222222222 & \\ = & A\;2\;(A\;2\;2)\;2 & 22222A2222222222 & \\ = & 2\;2\;(A\;2\;2) & 222222A2222222222 & \\ = & 4\;(A\;2\;2)\;2 & 22222A2222222222 & \\ = & A\;2\;2\;(A\;2\;2\;(A\;2\;2\;(A\;2\;2\;2))) & 22222A2222222222 & \\ = & A\;2\;2\;(A\;2\;2\;(A\;2\;2\;2)) & 22222222A2222222222 & \\ = & A\;2\;2\;(A\;2\;2\;2) & 22222222222A2222222222 & \\ = & A\;2\;2\;2 & 22222222222222A2222222222 & \\ = & 2\;2\;2\;2 & 22222222222222A2222222222 & \\ = & 2↑↑18\;A\;2\;2\;2\;2\;2\;2\;2\;2\;2\;2 & & \end{array}$□

These 2↑↑18 iterations of A let us relate its magnitude to the Fast Growing Hierarchy, a family of ordinal indexed functions [α] from natural numbers to natural numbers.

**Definition** **1.** *The (slightly accelerated) Fast Growing Hierarchy (FGH) below $\omega^{\omega }$, mapping Church numerals to Church numerals, is defined as*

-
$\left[0\right]\;n=2\;n=n^{2}$

-
[α+1]n=n2[α]2=A2[α]n

-
$\left[\omega^{i+1}\left(\alpha +1\right)\right]\;n=\left[\omega^{i+1}\alpha +\omega^{i}n\right]\;2$

We deviated from the standard FGH’s [0]n=n+1 and [α+1]n=n[α]n to make the following Lemma exact rather than a mere lower bound.

**Lemma** **5.** *For $k\geq 0,n\geq 2:\left(k+1\right)\;A\;2\;\left[\omega^{k}\alpha \right]\;n=\left[\omega^{k}\left(\alpha +1\right)\right]\;n$*.

**Proof.**  Base k=0: $\left(0+1\right)\;A\;2\;\left[\omega^{0}\alpha \right]\;n=A\;2\;\left[\alpha \right]\;n=n\;2\;\left[\alpha \right]\;2=\left[\alpha +1\right]\;n$.

Step k>0: $\left(k+1\right)\;A\;2\;\left[\omega^{k}\alpha \right]\;n=A\;\left(k\;A\;2\right)\;\left[\omega^{k}\alpha \right]\;n=n\;\left(k\;A\;2\right)\;\left[\omega^{k}\alpha \right]\;2=\left[\omega^{k}\alpha +\omega^{k-1}n\right]\;2=\left[\omega^{k}\left(\alpha +1\right)\right]\;n$. □

Lemmas 4 and 5 give $\omega \;218=2↑↑18\;A\;2\;\left[0\right]\;2\;2\;2\;2\;2\;2\;2\;2=2^{2^{2^{2^{2^{2^{2^{\left(\left[\omega^{2↑↑18-1}\right]\;2\right)}}}}}}}$. In comparison, Graham’s and Melo’s Numbers are known to be much smaller at around [ω+1]64 and [ω+1](2↑↑6) respectively.

## 9. A Functional Busy Beaver

The λ-calculus analogue to BB is as follows.

**Definition** **2.** *BBλ(n)= the maximum beta normal form size of any closed lambda term of size n*.

This appears in the Online Encyclopedia of Integer Sequences (OEIS) as sequence A333479. Besides being simpler than BB, it has the advantage of using the standard unit of information theory, bits rather than states.

The much more fine-grained use of bits allows the first 36 values of BBλ to be currently known, versus only five values of BB.

As a Church numeral, term Melo implies that BBλ(49)≥5(Melo’s Number)+6, and ω218 implies a similar lower bound on BBλ(61).

## 10. BB Compared Bit-by-Bit to BBλ

The growth rates of the TM-based BB function and the lambda calculus-based BBλ may be compared by how quickly they are known to reach certain ordinal milestones in the Fast Growing Hierarchy. We informally say that some huge number *N* reaches ordinal α if α is the least ordinal whose corresponding natural number function [α] in the FGH is sufficiently fast growing to comfortably express an upper bound on *N*. This is to say that *N* is exceeded by [α]n for a moderately small value of *n*, while exceeding *N* with any $[\alpha^{\prime }<\alpha ]n$ would require a huge value of *n* itself.

Lambda terms for the sizes given below can be found at the bbchallenge webpage [8].

For Graham’s Number, at ordinal ω+1, we saw earlier that Melo’s 49 bits compares with 14 states, which take 14×2×(2+4)=168 bits to encode. If I lose my bet, then the comparison becomes rather closer at 49 vs. 70 bits.

For Goodstein’s function, at ordinal $\epsilon_{0}$, 111 bits [9] compares with 51 states [10] taking 51×2×(2+6)=816 bits.

The $\epsilon_{0}$ growth term is actually obsoleted by a 100-bit [11] term, recently discovered by Patcail, that grows at the unfathomably larger Bucholz’ Ordinal, the catching point between the Slow Growing Hierarchy (SGH) and the FGH. As such, that term easily exceeds another famously large number, TREE(3).

For the limit of Bashicu Matrix System (BMS), at (presumed) ordinal PTO$\left(Z_{2}\right)$, 331 bits [12] compares with 150 states [10] taking 150×2×(2+8)=3000 bits.

Finally, for Loader’s Number, at (presumed) ordinal PTO$\left(Z_{\omega }\right)$, 1850 bits [13] compares with 1015 states [14] taking 1015×2×(2+10)=24360 bits.

One reason for TMs taking many more bits to achieve comparable growth, especially at the larger milestones, is the extremely poor programmability of TMs. The λ-calculus, despite its similar bare bones nature, does not share this drawback. Modern high-level pure functional languages like Haskell are essentially just syntactically sugared λ-calculus, with programmer friendly features like Algebraic Data Types translating directly through Scott encodings. The “bruijn” programming language is an even thinner layer of syntactic sugar for the pure untyped lambda calculus, whose extensive standard library contains many datatypes and functions. It is this excellent programmability of the λ-calculus that facilitated the construction of highly optimized programs for BMS and Loader.

Because programming a Turing machine is so impossibly tedious, people have resorted to implementing higher-level languages like Not-Quite-Laconic for writing nontrivial programs, such as the TM that halts only upon finding an inconsistency in ZFC. The above 1015 state TM for exceeding Loader’s Number even includes a λ-calculus interpreter written in NQL in order to emulate the BBλ champion.

## 11. But Why Turing Machines?

In his paper [15] “The Busy Beaver Frontier”, Scott Aaronson poses and tries to answer this question:

For all their historic importance, haven’t Turing machines been completely superseded by better alternatives—whether stylized assembly languages or various codegolf languages or Lisp? As we’ll see, there is a reason why Turing machines were a slightly unfortunate choice for the Busy Beaver game: namely, the loss incurred when we encode a state transition table by a string of bits or vice versa.

He continues to point out two supposedly massive advantages that TMs have over alternatives, which we can assess for the case of the λ-calculus.

### 11.1. Interesting Behavior at Small Sizes

Aaronson continues:

because Turing machines have no “syntax” to speak of, but only graph structure, we immediately start seeing interesting behavior even with machines of only 3, 4, or 5 states, which are feasible to enumerate.

The number of uniquely behaving TMs with 5 states is $4^{10}\times 632700=$ 663,434,035,200 (see OEIS sequence A107668), which is more than the number of closed lambda terms of size at most 52 bits (513,217,604,750). The latter exhibits just as much interesting behavior, so TMs hold little advantage here.

### 11.2. Ancient and Fixed Computational Model

Aaronson ends his answer with:

because the Turing machine model is so ancient and fixed, whatever emergent behavior we find in the Busy Beaver game, there can be no suspicion that we cheated by changing the model until we got the results we wanted.

The λ-calculus is slightly more ancient and arguably more fixed, without any of the TM’s choices: tape alphabet size, whether the tape head moves in every transition, halting and output convention, number of tapes or tape heads. Both TMs and the λ-calculus are maximally non-cheating, so again TMs hold little advantage here.

The only remaining advantage of BB over BBλ is the many decades of research behind, and publications about it.

Is BBλ then an ideal Busy Beaver function? Not quite. Andreev [16] rightly criticizes BB for being too dependent on the Turing machine model, with small changes in the model yielding different growth rates. Similarly, BBλ is too dependent on the lambda calculus and its binary encoding. Grounding a busy beaver in Algorithmic Information Theory (AIT) instead yields a more invariant approach.

## 12. Kolmogorov Complexity

The main object of study in AIT is the (plain) Kolmogorov complexity C(x) of a string *x*, defined as the length of a shortest description of *x*, i.e., a program that halts with output *x*. While that seems to define a different complexity measure for every possible programming language, the Invarariance theorem guarantees the existence of an additively optimal language $L_{opt}$, one that offers descriptions at most a constant longer than those in any other language: $\forall L\exists c\forall x:C_{L_{opt}}\left(x\right)\leq C_{L}\left(x\right)+c$.

Li and Vitányi’s standard reference [17] proves it in Theorem 2.1.1 for Turing machines with inputs delimited by blanks, and in Theorem 3.1.1 for self-delimiting machines, which lack a blank tape symbol and must therefore determine when to stop reading input bits based only on what bits have been read so far. It follows that different optimal languages yield complexities differing by at most a constant.

AIT typically proceeds with assuming an arbitrary but fixed optimal (also called universal) language, and then studying properties of C() and its self-delimiting counterpart K() expressed with undetermined O(1) constants. For example, the basic result that complexity of a binary string is, up to a constant, at most its length: C(x)≤l(x)+O(1). None of these results need to be concerned with cheating, as any cheating benefits get absorbed by the O(1) terms.

I designed the Binary Lambda Calculus (BLC) programming language [5] to make Kolmogorov complexity more concrete, following in the footsteps of Gregory Chaitin [18] with his custom LISP languages. A BLC program consists of the binary encoding of a lambda term *L*, followed by arbitrary binary data $s\in {\left\{0,1\right\}}^{*}$. For the data to be operated on, it must be represented as a lambda term itself, along with a choice of whether it is delimited. A bit *b* is represented as $\lambda x_{0}\lambda x_{1}.x_{b}$, and a sequence is represented by nested pairing with the pairing function cons=λxλyλz.zxy. The term representing *s* delimited by some delimiter term del is denoted (s:del).

The Invariance theorem for BLC is proven with the universal lambda machine term *U* defined in Section 4.1 of [5], which satisfies $U(\hat{L}:N)=L\;N$ for all terms *N*. By appropriate choice of input delimiter, one can thus concretely define the plain Kolmogorov complexity function and its self-delimiting counterpart:

KS(x)=min{l(p)|U(p:nil)=x}

KP(x)=min{l(p)|U(p:Ω)=x}

Here, nil=λxλy.y provides delimited input, while Ω=(λx.xx)(λx.xx) is a self-loop enforcing self-delimiting behavior.

We can now prove concrete theorems like KS(x)≤l(x)+4, where 4 is the size of the identity function λx.x.

## 13. An Optimal Busy Beaver

The same notion of invariance/optimality can be applied to busy beavers. An optimal busy beaver BB_opt_ should surpass any busy beaver function bb with at most a constant increase in program size:


$$\forall bb\exists c\forall n:{\mathrm{BB}}_{\mathrm{opt}}\left(n+c\right)\geq bb\left(n\right)$$

Just like any closed lambda term *L* defines a description language, so it defines a busy beaver. Let a self-delimiting halting *L*-program be any binary string *s* such that L(s:Ω) has a normal form but $L(s^{\prime }:\Omega )$ has no normal form for any proper prefix $s^{\prime }$ of *s*. This means that *L* is “reading” all the bits of *s* before halting.

**Definition** **3.** *For any closed lambda term L, define its corresponding busy beaver function as $bb_{L}\left(n\right)=$ the maximum normal form size of any halting self-delimiting L-program of length n (or 0 if there is none). Define BB$\lambda 2\left(n\right)=bb_{U}$*.

**Theorem** **2.** *BBλ2 is optimal.*

The proof is almost identical to the Invariance theorem for BLC.

**Proof.**  Since U satisfies $U(\hat{L}:N)=L\;N$ for all terms *N*, we get BB$\lambda 2\left(n+l\left(\hat{L}\right)\right)\geq bb_{L}\left(n\right)$. □

BBλ2 appears as sequence A361211 in the OEIS.

## 14. An Inverse Kolmogorov Complexity

Instead of Definition 3, one can simply define a busy beaver as an inverse of Kolmogorov complexity, B(n)=max{x∈N:C(x)≤n}, as Vereschagin et al. did in [19], and which Andreev followed in [16]. They also defined BB(n) as the maximal runtime of delimited programs of length at most *n*, and BP(n) as the maximal runtime of self-delimited programs of length at most *n*. Both papers study the asymptotic properties of these functions.

BBλ2 is almost an inverse complexity. Consider the following variant whose definition resembles B():

**Definition** **4.** *Let NF be the set of closed terms in normal form. BB$\lambda 2^{\prime }\left(n\right)=\max\left\{0\right\}\cup \left\{l\left(\hat{x}\right):x\in NF\wedge KP\left(x\right)\leq n\right\}$*.

Just like B(), this function is necessarily nondecreasing. But BBλ2 is not. For instance BBλ2(28)=BBλ(26)=52>BBλ2(29)=BBλ(27)=44.

Since BB$\lambda 2^{\prime }\left(n\right)$ is the maximum normal form size of terms with complexity at most *n*, it is the maximum over all $n^{\prime }\leq n$ of (the maximum normal form size of terms with a BLC program of size $n^{\prime }$), with the latter being exactly BB$\lambda 2(n^{\prime })$. This proves

**Theorem** **3.** *BB$\lambda 2^{\prime }\left(n\right)=\max\left\{BB\lambda 2\left(n^{\prime }\right):n^{\prime }\leq n\right\}$,*

It makes sense to prefer the nondecreasing version for its nicer asymptotic properties, but for computing concrete Busy Beaver values, having more champions is to be preferred.

## 15. BBλ vs. BBλ2

Since (λ_.t), applied to any binary data term, equals *t*, BBλ champions provide lower bounds for BBλ2: for all *n*, BBλ2(2+n)≥BBλ(n).

In BBλ2 and BBλ(n), we thus have a moderately simple optimal Busy Beaver function and a really simple one that closely approximates it for all the smaller values of *n* that we can hope to analyze.

In the coming years we can look forward to many more values of BBλ(n) being determined beyond the first 37. As with BB, this effort promises to generate an interesting set of open mathematical problems that challenge the limits of mathematical knowledge.

## 16. Conclusions

The largest number (currently known to be) representable in 64 bits without cheating is ω218, which lower bounds both BBλ(61) and BBλ2(63), with BBλ2 being the first instance of a concretely defined optimal Busy Beaver.

## Data Availability

Data are contained within the article.
